# Development of a pharmacovigilance safety monitoring tool for the rollout of single low-dose primaquine and artemether-lumefantrine to treat *Plasmodium falciparum* infections in Swaziland: a pilot study

**DOI:** 10.1186/s12936-016-1410-7

**Published:** 2016-07-22

**Authors:** Eugenie Poirot, Adam Soble, Nyasatu Ntshalintshali, Asen Mwandemele, Nomcebo Mkhonta, Calisile Malambe, Sibonakaliso Vilakati, Sisi Pan, Sarah Darteh, Gugu Maphalala, Joelle Brown, Jimee Hwang, Cheryl Pace, Andy Stergachis, Eric Vittinghoff, Simon Kunene, Roland Gosling

**Affiliations:** Global Health Group, University of California San Francisco, San Francisco, CA USA; Department of Epidemiology and Biostatistics, University of California San Francisco, San Francisco, CA USA; Clinton Health Access Initiative, Mbabane, Swaziland; University of Namibia, Windhoek, Namibia; National Malaria Control Programme, Manzini, Swaziland; International Center for AIDS Care and Treatment Programs, Mbabane, Swaziland; Swaziland Health Laboratory Services, Mbabane, Swaziland; President’s Malaria Initiative, Malaria Branch, U.S. Centers for Disease Control and Prevention, Atlanta, GA USA; Liverpool School of Tropical Medicine, Liverpool, UK; Departments of Pharmacy and Global Health, Schools of Pharmacy and Public Health, University of Washington, Seattle, USA

**Keywords:** Elimination, Glucose-6-phosphate dehydrogenase deficiency, G6PD, Malaria, Pharmacovigilance, *Plasmodium falciparum*, Primaquine, Safety

## Abstract

**Background:**

Countries remain reluctant to adopt the 2012 World Health Organization recommendation for single low-dose (0.25 mg/kg) primaquine (SLD PQ) for *Plasmodium falciparum* transmission-blocking due to concerns over drug-related haemolysis risk, especially among glucose-6-phosphate dehydrogenase-deficient (G6PDd) people, without evidence demonstrating that it can be safely deployed in their settings. Pharmacovigilance methods provide a systematic way of collecting safety data and supporting the rollout of SLD PQ.

**Methods:**

The Primaquine Roll Out Monitoring Pharmacovigilance Tool (PROMPT), comprising: (1) a standardized form to support the surveillance of possible adverse events following SLD PQ treatment; (2) a patient information card to enhance awareness of known adverse drug reactions of SLD PQ use; and (3) a database compiling recorded information, was developed and piloted. Data on patient characteristics, malaria diagnosis and treatment are collected. Blood samples are taken to measure haemoglobin (Hb) and test for G6PD deficiency. Active follow-up includes a repeat Hb measurement and adverse event monitoring on or near day 7. A 13-month prospective pilot study in two hospital facilities in Swaziland alongside the introduction of SLD PQ generated preliminary evidence on the feasibility and acceptability of PROMPT.

**Results:**

PROMPT was well received by nurses as a simple, pragmatic approach to active surveillance of SLD PQ safety data. Of the 102 patients enrolled and administered SLD PQ, none were G6PDd. 93 (91.2 %) returned on or near day 7 for follow-up. Four (4.6 %) patients had falls in Hb ≥25 % from baseline, none of whom presented with signs or symptoms of anaemia. No patient’s Hb fell below 7 g/dL and none required a blood transfusion. Of the 11 (11 %) patients who reported an adverse event over the study period, three were considered serious and included two deaths and one hospitalization; none were causally related to SLD PQ. Four non-serious adverse events were considered definitely, probably, or possibly related to SLD PQ.

**Conclusion:**

Improved pharmacovigilance to monitor and promote the safety of the WHO recommendation is needed. The successful application of PROMPT demonstrates its potential as an important tool to rapidly generate locally acquired safety data and support pharmacovigilance in resource-limited settings.

**Electronic supplementary material:**

The online version of this article (doi:10.1186/s12936-016-1410-7) contains supplementary material, which is available to authorized users.

## Background

As malaria programmes move towards elimination, stopping onward transmission from all detected cases becomes increasingly important. The most commonly used anti-malarials are artemisinin derivatives, which destroy early developing *Plasmodium falciparum* gametocytes (stages I to III) and asexual blood stages of human malaria species [1]. However, most symptomatic cases present with measurable and transmissible levels of mature gametocytes (stages IV and V), as determined by Pfs25 RNA-based quantitative nucleic acid sequence-based amplification (Pfs25 QT-NASBA), which can infect mosquitoes, even after completing artemisinin-based combination therapy (ACT) [2–4]. The 8-aminoquinoline compounds are the only class of drugs effective against mature *P. falciparum* gametocytes. Primaquine (PQ) is currently the only licensed anti-malarial in this class of drugs.

Since its approval by the FDA in 1952, PQ has been used as 14-day anti-relapse treatment against *Plasmodium vivax* and *Plasmodium ovale* species [5, 6]. In addition, in combination with an effective ACT, a single dose of PQ can prevent onward transmission of *P. falciparum* [1, 7]. However, PQ can cause haemolysis in people who are deficient in glucose-6-phosphate dehydrogenase (G6PD) [8]. The extent of an individual’s susceptibility to and severity of PQ-induced haemolysis depends on dose and duration of PQ exposure, and degree of G6PD deficiency [8–11].

Previously, the World Health Organization (WHO) recommended the addition of a single 0.75 mg/kg dose of PQ to ACT for the clearance of *P. falciparum* gametocytes. However, low uptake of this recommendation and concerns over the potential for haemolytic toxicity in G6PD-deficient (G6PDd) individuals prompted the issue of a new WHO directive, recommending the use of a lower 0.25 mg/kg dose of PQ in areas threatened by artemisinin resistance and in settings targeting malaria elimination. This 0.25 mg/kg dose should be given to all patients with parasitologically-confirmed *P. falciparum* malaria together with ACT, except for pregnant women and infants. Prior G6PD testing is not required [12]. Despite evidence suggesting that the risks for haemolysis are low at the 0.25 mg/kg PQ dose, it is a common concern that PQ, even at low doses, may result in severe haemolysis in G6PDd individuals [13]. There remains limited data to suggest the safety of this recommendation and countries have been reluctant to implement the policy without more evidence demonstrating that it can be safely deployed in their setting.

The use of standardized, prospective pharmacovigilance methods for PQ provides an opportunity to generate data that can be used to confirm or refute safety concerns. To date, pharmacovigilance on PQ is weak and relies predominately on passive reporting. In most countries in sub-Saharan Africa, passive reporting systems, which lack an appropriate denominator of persons exposed to treatment, are limited or non-existent [14]. From a total of 1429 reports (4560 events) submitted to the Uppsala Monitoring Center, the WHO’s international drug monitoring programme, in which PQ was suspected to be a causative or interacting factor for the event, 89 % were reported from Thailand and only one report (3 events) came from Africa [15]. Monitoring the use of PQ offers a mechanism to more accurately quantify the risk of haemolysis associated with PQ administration, especially in patients who are G6PDd. Unlike other anti-malarial drug regimens where adverse drug reactions may be unexpected or poorly defined, requiring weeks of follow-up, the objectives for monitoring PQ use are straightforward; signs of haemolytic anaemia, the main safety concern, can be more specific (i.e., dark urine), easily measurable by following haemoglobin concentration, and attainable within a week of drug administration through active surveillance methods.

To support the safe rollout of SLD PQ, the Primaquine Roll Out Monitoring Pharmacovigilance Tool (PROMPT) was developed in collaboration with the National Malaria Control Programme (NMCP) in Swaziland. The objective of this study was to assess the feasibility and perceived acceptability of using a simple pharmacovigilance tool to collect standardized safety data in those prescribed SLD PQ therapy for the treatment of *P. falciparum* malaria.

## Methods

This project involved two phases: (1) a tool development phase during which the components of PROMPT were developed and (2) a pilot phase, when intended end-users in Swaziland participated in testing PROMPT alongside the introduction of SLD PQ.

### Tool development

#### Tools

Through an interactive process, involving a literature review and discussions with national and international experts, a tool was developed comprising three parts: (1) a standardized form (on either a paper or electronic platform) collecting a minimum set of essential data elements to support the surveillance of possible adverse events following SLD PQ treatment; (2) a patient information card to enhance awareness of known adverse drug reactions of SLD PQ use; and (3) a database compiling recorded information.

##### Data collection form

Data collection begins once the prescriber has decided to treat confirmed, uncomplicated malaria cases with PQ (day 0), in addition to the standard blood schizonticide recommended by that particular country. The prescriber collects basic socio-demographic data (age, sex, and weight), contact information, patient and family history data (e.g., severe anaemia, G6PD deficiency, blood transfusions), malaria diagnosis information, and drug dosage and frequency data.

For those involved with active surveillance, a blood sample is collected to measure haemoglobin or haematocrit and to screen for G6PD deficiency. Patients are asked to return for a scheduled follow-up visit on or near day 7 because drug-induced hemolysis and jaundice are clinically detectable within the first 7 days [8]. At this time, a repeat haemoglobin or haematocrit measure is taken to gauge the degree and speed of decline from the baseline value prior to starting PQ. Information is also gathered about possible adverse events and serious adverse events, defined according to International Conference of Harmonization guidelines [16]. The form comprises a short list of possible events (e.g., nausea, vomiting, dark urine, diarrhoea, etc.) as well as space for recording any other signs or symptoms external to the list provided. Specifically, data on all new onset or worsening signs or symptoms, following PQ treatment (i.e., treatment emergent adverse events), regardless of causality, are collected. Patients involved with active monitoring who miss their day 7 assessments are phoned within a week to ask about adverse events. All adverse events reported as part of passive or active follow-up record the following information: description of the adverse event, date of onset, severity grade, actions taken, its relatedness to PQ and/or the blood schizonticide used, date resolved, outcome of adverse event, and other drugs (including traditional medicines) taken along with PQ at the time of treatment and in the two-week period prior to the onset of the event. Severity grades follow the WHO toxicity grading scale for determining the severity of adverse events [17]. In addition, for those presenting with suspected PQ-induced haemolysis, urine colour is recorded using a Hillmen colour chart designed to assess the presence of haemoglobinuria (Additional file 1) [18]. A colourimetric level of 5 or above is considered evidence of haemoglobinuria.

##### Patient information card

At the time of prescribing PQ (day 0), patients are given a patient information card with oral and written instructions on how to identify the signs and symptoms of known adverse drug reactions of PQ (e.g., gastrointestinal upset, dark urine, backaches, etc.) and to monitor the colour of their urine (Additional file 2). The card also contains instructions of what to do and a telephone number to call if adverse drug reactions are experienced. Patients are instructed to immediately return to the clinic should they experience any adverse reactions following PQ treatment.

##### Database

Data from health facilities are uploaded into a secure country-level database. In settings where electronic data collection is feasible, the database can assist with managing follow-up visits and remind study participants and caregivers of follow-up appointments, including producing a telephone follow-up call list. Anonymized data from end-users can then be fed to a global database where data can be combined and analysed.

#### Sample size considerations

Sample size calculations were based on two scenarios for detecting a 25 % or greater within-in person reduction in haemoglobin after PQ treatment in G6PDd persons using a one-sample paired t-test with a 0.05 level of significance (one-sided). A 25 % or greater reduction was regarded as the minimum clinically important threshold that would signify caution for implementing the addition of SLD PQ to ACT for the treatment of *P. falciparum* infections. Under the first scenario, in programme settings where G6PD screening is available at the individual level, seven G6PDd individuals would provide 80 % power to detect a 25 % or greater within-person reduction in haemoglobin, assuming a mean haemoglobin of 10 g/dL before treatment, a standard deviation of 2 g/dL for the within-person change in haemoglobin, and up to 25 % loss to follow-up. In the second scenario, in programme settings where G6PD screening is unavailable and status is unknown, a programme with an estimated 10 % population prevalence of G6PD deficiency would, under the same assumptions, need to treat a minimum of 70 individuals with SLD PQ (Additional file 3).

### Pilot phase

#### Study area

In response to a request from the Ministry of Health (MoH), PROMPT was first piloted in the Lubombo and Manzini regions of the Kingdom of Swaziland alongside the introduction of SLD PQ. Good Shepherd Mission Hospital, a rural, regional referral hospital in Lubombo, and Raleigh Fitkin Memorial Hospital, a hospital in Manzini run by the Catholic mission and government were both purposively selected by the NMCP to represent facilities with the highest burden of malaria cases. Lubombo and Manzini reported a total of 266 and 164 malaria cases, respectively, this past year (March 2014–April 2015) [19]. Malaria transmission is highest in the lowlands of the Lubombo region and peaks in the rainy season between November and May. *Plasmodium falciparum* is the predominant parasite species accounting for over 99 % of malaria cases. One-third of Swaziland’s population is at risk of infection, with residents in the Lubombo region near the eastern borders with Mozambique and South Africa at greatest risk.

#### Pre-piloting

##### Stakeholder engagement and workflow integration

The pilot was tested following discussions with key stakeholders, including the MoH, an experienced database developer, infectious disease specialists with pharmacovigilance expertise, and local health workers (nurses, laboratory technologists, nurse matrons and doctors) over 6 months. Consultations with the database developer allowed us to build a user-friendly, electronic data collection tool configured to country capacity. Infectious disease and pharmacovigilance specialists offered guidance on how to standardize and define the data elements (and minimum reporting criteria) collected. Health workers reviewed the steps necessary to use PROMPT and increased awareness around its use. Observations were made at each facility and discussions were conducted with clinic staff to best integrate PROMPT into the existing clinical workflow and capture confirmed malaria cases.

##### Training for the pilot evaluation

Nurses from both facilities received a two-day training workshop followed by 7 days of pre-pilot on-site training. Staff from the NMCP, health facility doctors and nurse matrons were also invited to the group training to enable additional support to trained health workers during study implementation. Half way through study implementation (August 2014) trained health workers received a 1-day refresher training. Both trainings focused on illustrating routine study procedures. The health workers were trained in data reporting procedures and electronic data collection. Each facility received job aids (e.g., flowcharts and figures) to offer cues and direction on key procedures. Instruction on identifying the signs and symptoms of acute haemolytic anaemia, the main adverse effect of SLD PQ use, was also given. Training objectives also included opportunities for the study team to complete tool procedures in a pre-testing environment and to solicit feedback from the team on components of PROMPT needing further refinement.

#### Pilot-testing PROMPT

##### Recruitment

All patients over 1 year of age and prescribed standard doses of first-line artemether-lumefantrine (AL) for confirmed, uncomplicated *P. falciparum* malaria were eligible to receive SLD PQ. Patients were not deemed suitable for SLD PQ treatment if they had any of the following: evidence of severe malaria [20]; history of allergies to study drugs; acute anaemia, defined as haemoglobin <8 g/dL; were pregnant by pregnancy test among women of childbearing age (15–49 years) or breastfeeding. Patients selected for inclusion also had to agree to follow-up phone calls and/or visits and had no intentions of leaving Swaziland for a 14-day follow-up period.

##### Procedures

Prior to SLD PQ administration, G6PD deficiency screening was performed using the CareStart G6PD deficiency screening kit (Access Bio. Inc., New Jersey, USA, LOT No. GP13E01; GP13C01) and haemoglobin was measured using HaemoCue 201+ photometers (HaemoCue, Angelholm, Sweden). Patients were then treated with the WHO recommended single dose of PQ (0.25 mg/kg) (Primaquine^®^, Micro Labs Ltd Hosur, Bangalore, India) using 7.5 mg PQ tablets, according to four weight bands established following discussions with country doctors and senior officials from the NMCP (Table 1). Primaquine tablets were analysed by high-performance liquid chromatography (U.S. Centers for Disease Control and Prevention, Atlanta, GA, USA) to measure the concentration of active pharmaceutical ingredient (API) against a reference standard; tested tablets possessed over 95 % API. Patients were given SLD PQ with the first dose of AL together with food and then directly observed for 30 min. If a patient vomited within 30 min, treatment was re-administered. Those who vomited a second time were excluded from further SLD PQ dosing.

**Table 1:** Dosage chart for single low-dose primaquine

Weight (kg)	Number of tablets	Dose (mg)	Dose range (mg/kg)
10–15	0.5	3.75	0.375–0.25
16–30	1	7.5	0.469–0.25
31–45	1.5	11.25	0.363–0.25
>45	2	15	<0.333

At the time of prescribing SLD PQ, trained nurses collected data as outlined in the data collection form and provided patients with a patient information card. Patients were scheduled for follow-up visits on or near day 7 and received SMS text message reminders 2 days prior to scheduled visits and on day 7. Patients were also encouraged to follow-up at unscheduled times should they feel unwell. Patients were asked general open-ended questions to elicit events and to determine if they had experienced any problems since being treated with SLD PQ. Responses were probed to solicit further details on reported events. All adverse and serious adverse events reported by participants were examined by hospital physicians and recorded by trained nurses. If medically indicated, patients were closely monitored and appropriate referrals were made according to national protocol. Patients were telephoned within a week of missed visits to collect information on adverse events. Reporting of adverse events followed local ethics and UCSF Institutional Review Board guidelines.

**Fig. 1:**
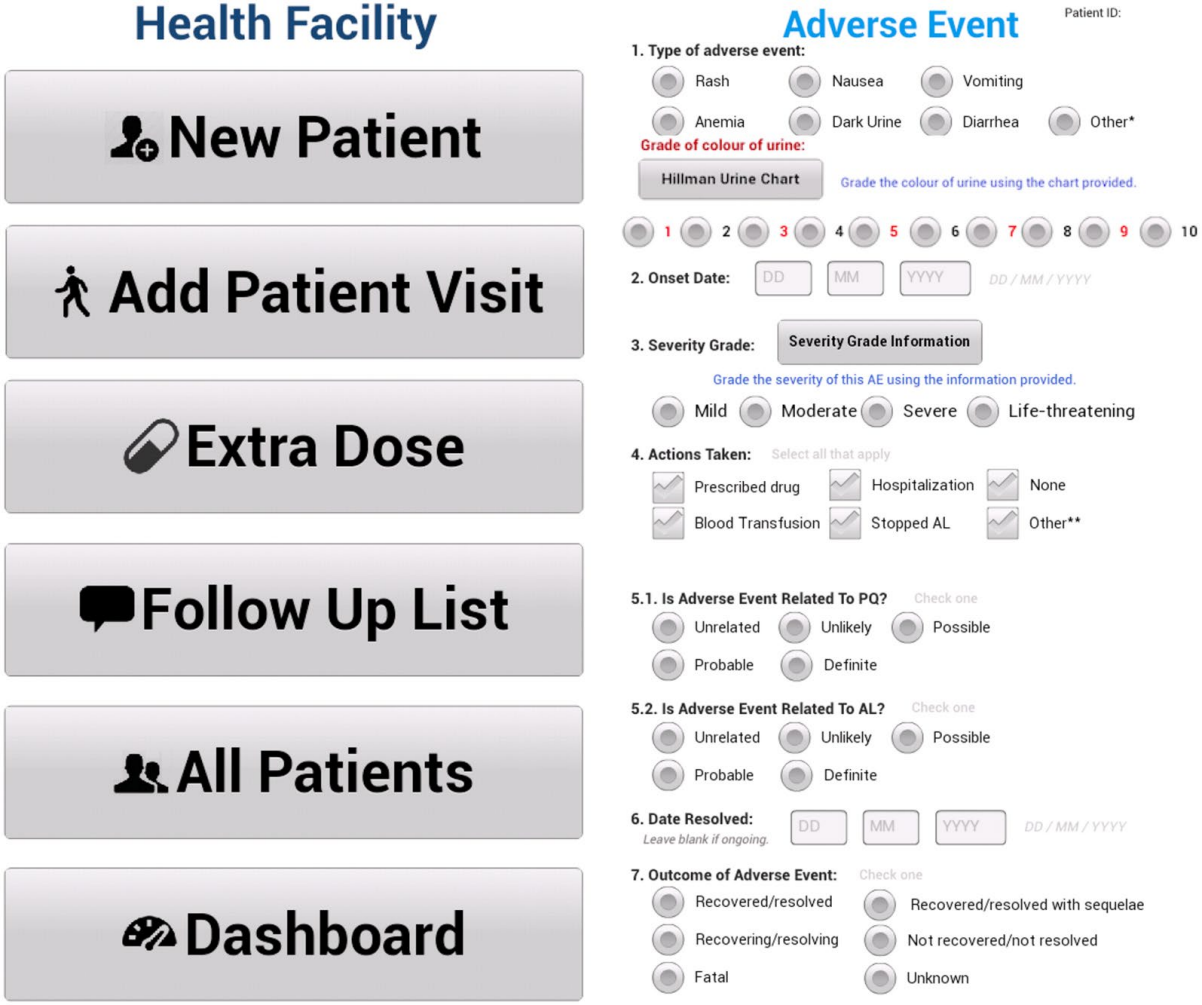
Screenshot images of data collected using Samsung T211 Galaxy Tab 3 (7.0) 3G tablets

Questionnaire data from each visit were entered into Samsung T211 Galaxy Tab 3 (7.0) 3G (Samsung, Seoul, Korea) tablets running Android OS to enable automated data collection in the health facilities (Fig. 1). The tablets were synchronized directly to a secure country-level database for real-time review. The data were checked by a co-investigator for inconsistencies and corrected by contacting the study nurses. The database also served to manage follow-up visits and remind subjects and caregivers of follow-up appointments via automated reminders using SMS text messaging. In addition, the database produced a telephone follow-up call list for nurses, accessible directly from the tablets, to track and follow-up with patients who missed their scheduled visits.

### Data analysis plan

#### Outcome measures and definitions

##### Tool evaluation—feasibility and perceptions of acceptability

To assess feasibility, the proportion enrolled among those eligible and the proportion of patients completing study procedures, looking closely at day 7 follow-up adherence, were measured. Process measures (e.g., visit length, provider attitudes about clinic burden, satisfaction with counselling sessions with patients, etc.) and reactions to and ease or challenges of study procedures were collected, using a semi-structured questionnaire, to investigate perceptions related to acceptability among trained nurses. Discussions (one per hospital facility) took about 60 min to complete. A moderator collected handwritten or electronic notes.

##### Safety assessment of single low-dose primaquine

To assess the safety of introducing SLD PQ, the haematologic response and occurrence of adverse events in those receiving SLD PQ therapy were investigated. Haemoglobin concentrations (defined in g/dL) were expressed as the absolute and relative change from baseline values (day 0). Anaemia was classified according to WHO guidelines [21] (6–59 months Hb <11 g/dL; 5–11 years Hb <11.5 g/dL; 12–14 years Hb <12.0 g/dL; ≥15 years, non-pregnant women Hb <12.0 g/dL; pregnant women Hb <11.0 g/dL; ≥15 years men Hb <13.0 g/dL).

#### Data analysis

Descriptive statistics were used to present the demographic characteristics of the study population and their baseline clinical and biological characteristics. Data were presented in terms of mean and standard deviations or median and interquartile range for continuous variables and percentages for categorical variables. All patients with complete haemoglobin values who returned for follow-up within 10 days after treatment were included in assessments of haematological changes. Categorical data were compared using the Chi square test or a Fisher’s exact test, when applicable. Analyses of reported adverse events were based on descriptive summaries of frequency, severity, and relatedness to SLD PQ. Quantitative data were analysed using STATA 13.0 (© StataCorp, College Station, TX, USA). Qualitative data from interview notes were summarized after familiarization of the data using an iterative approach. Emergent themes from the data were identified based on frequency of appearance and summarized.

### Ethical considerations

The study was approved by review committees at the Swaziland MoH (reference MH/599C/FWA 000 15267) and the University of California, San Francisco (IRB no. 13-11626), and granted non-engaged status by the United States Centers for Disease Control and Prevention. Participants or a parent or guardian for children less than 7 years of age gave written, informed consent; children between the ages of 7 and 17 years gave assent following national guidelines.

## Results

### Feasibility

**Fig. 2:**
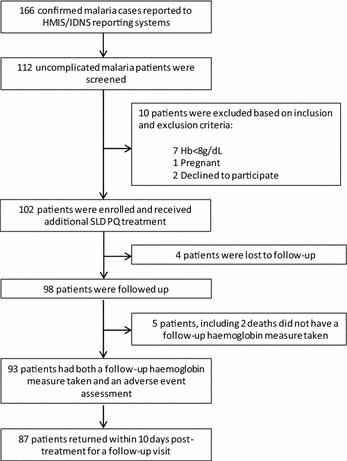
Cohort profile

Over a 13-month pilot period (March 2014–April 2015), 166 confirmed, uncomplicated malaria cases from two hospital facilities in Lubombo (77) and Manzini (89) regions were reported through Swaziland’s Health Management Information System and Immediate Disease Notification System. Of those reported, 112/166 (67.5 %) patients were seen by four nurses trained in the use of PROMPT and screened for inclusion. Ten patients were excluded: one tested positive for pregnancy, seven had haemoglobin concentrations less than 8 g/dL, and two declined to participate. A total of 102 patients (59 from Lubombo and 43 from Manzini) were enrolled and administered SLD PQ and 98/102 (96.0. %) were followed up with 93/102 (91.2 %) successfully completing all follow-up procedures. This included obtaining a repeat haemoglobin measurement and collecting data on all adverse events experienced since taking SLD PQ (Fig. 2). Four patients (3.9 %) were lost to follow-up with no data collected after taking SLD PQ at the health facility. Demographic characteristics did not differ significantly between those who were lost to follow-up and those who were retained, except for foreign nationality. Those lost to follow-up were more likely to be of foreign nationality.

Patients were scheduled to return on or near day 7 for a follow-up visit; however, the timing of scheduled post-treatment follow-up visits varied. Patients returned for a follow-up visit as early as 4 days post-treatment to as late as 23 days post-treatment. Nonetheless, adherence to visits on or near day 7 was good, with 66/102 (64.7, 95 % CI: 54.6–73.9 %) returning promptly on day 7 and 87/102 (85.3, 95 % CI: 76.9–91.5 %) returning within 10 days (range: 4–10) post-treatment for a follow-up visit.

### Perceptions of acceptability

All four trained nurses participated in informal feedback discussions to offer initial perceptions of PROMPT’s acceptability and to suggest ways to improve upon the existing tool. The standardized data collection form using handheld electronic devices was popular. Nurses noted several benefits of using an electronic data entry system over paper for monitoring the safety of SLD PQ. All nurses agreed that this included the time saved on data entry and half of nurses felt that the electronic devices kept patient records confidential and accessible only by trained nurses and staff. There was also general agreement amongst the nurses that the average staff time required for screening and enrolment (27.5 min; range: 20–45 min), and follow-up visits (12.5 min; range: 10–15 min) per patient was acceptable. This period of time excludes the time taken for retrieving missing or additional information.

These discussions also revealed their views on counselling sessions with patients on G6PD deficiency and known adverse drug reactions associated with SLD PQ use. All nurses agreed that the patient information cards were a useful component of PROMPT that facilitated the performance of study procedures and conveyed key messages. However, nurses agreed that the language used to communicate G6PD deficiency and adverse effects of SLD PQ use to patients was challenging to develop. In addition, nurses highlighted challenges with the adverse event reporting process, including challenges with identifying cases as relevant to report. Nurses more frequently reported on events that were either severe or life-threatening in nature or expected signs and symptoms of SLD PQ use (i.e., dark-coloured urine, anaemia, jaundice).

The main challenge described when using an electronic platform was server connectivity; while nurses were still able to enter information and store data while the server was offline, data could not be synchronized and uploaded to the country-level database. This limited capacity to monitor and analyse data in real-time and resulted at times in delayed or duplicated SMS text messages being sent to patients.

### Baseline characteristics of participants enrolled in PROMPT

In our cohort of enrolled *P. falciparum* malaria patients treated with SLD PQ who completed follow-up (n = 93), the mean age was 25 years, ranging from 13 months to 68 years (Table 2). Overall, 17.2 % of participants were under 5 years of age. Anaemia was frequent before treatment: 26 patients (28.0, 95 % CI: 19.1–38.2 %) were mildly anaemic and 36 (38.7, 95 % CI: 28.8–49.4 %) were moderately anaemic. The majority of patients less than 15 years (n = 32) were anaemic at baseline (93.8, 95 % CI: 79.2–99.2 %). None of the enrolled patients were G6PDd at baseline (95 % upper confidence limit [UCL]: 3.6 %). One child screened for inclusion was G6PDd; however, the child was excluded and not given SLD PQ based on a haemoglobin concentration less than 8 g/dL.

**Table 2:** Baseline characteristics (n = 93)

Characteristic	% (n/N) or median (IQR)
Female	46 % (43/93)
Participants from Manzini district	59 % (55/93)
Age (years)	27 [5–38]
Weight (kg)	58 [20–71]
Baseline haemoglobin concentration (g/dL)	11 [10–13]

Data are presented as  % (n/N) or median [IQR]

*IQR* interquartile range

### Safety assessment of single low-dose primaquine

#### Haemolysis

Out of the 93 patients who successfully completed all follow-up procedures, 87 patients returned for at least one follow-up haemoglobin visit within 10 days of receiving SLD PQ with 58 (66.7 %) experiencing declines in haemoglobin concentration post-treatment. The mean absolute reduction in haemoglobin concentration within 10 days, relative to day 0 was 0.6 g/dL (95 % CI: −0.9–0.3 g/dL; p = 0.0008) (Table 3). The relative mean decrease in haemoglobin was 3.7 % (95 % CI: −6.4–1.0 %; p = 0.007). Few patients (11/87, 12.6 %) dropped 2 g/dL or more relative to day 0 (range: 2.1–5.9 g/dL). The maximum absolute decrease in haemoglobin concentration was 5.9 g/dL though this individual patient started with a high haemoglobin concentration of 20.1 g/dL that was likely due to dehydration at baseline. Four patients (4.6, 95 % CI: 1.3–11.4 %), all male adults with a starting haemoglobin concentration greater than 13 g/dL, experienced fractional drops in haemoglobin of 25 % or greater from baseline; the median absolute and relative drops in haemoglobin among these four patients were 4.9 g/dL and 30.5 %, respectively. Two patients (2.3, 95 % CI: 0.3–8.1 %) dropped below 8 g/dL; both were moderately anaemic at baseline. No patient who fell below 8 g/dL or experienced falls in haemoglobin of 25 % or greater post-treatment was clinically symptomatic with signs of anaemia or dark urine (macroscopic haemoglobinuria or Hillmen urine score ≥5). None of the patients dropped to a level below 7 g/dL (95 % UCL: 4.2 %).

**Table 3:** Baseline and follow-up values and changes in haemoglobin concentration within 10 days post-treatment in participants who received AL + SLD PQ (n = 87)

Characteristic	Mean (SD) (range)
Baseline haemoglobin (g/dL)	11.6 (2.3) [8–20.1]
Follow-up haemoglobin (g/dL)	11.0 (1.8) [7.2–15.8]
Absolute change in haemoglobin (g/dL)	−0.6 (1.6) [−5.9–3]
Percent change in haemoglobin (g/dL)	−3.7 (12.7) [−31.4–30.9]

Primaquine was given on day 0 of treatment, together with dose 1 of artemether-lumefantrine. Mean absolute change defined as the haemoglobin mean on the day of follow-up minus the haemoglobin mean at day 0. Mean relative percentage change between day 0 (t1) and follow-up (t2) defined as [(hb(t2)−hb(t1))/hb(t1)] × 100

*AL* artemether-lumefantrine, *Hb* haemoglobin, *SLD PQ* single low-dose primaquine, *SD* standard deviation

#### Description of reported adverse events

In general, SLD PQ was well tolerated by patients. Out of all the patients who received SLD PQ and returned for follow-up, 89 % (87/98) of patients did not report any adverse event since receiving treatment. Of the 11 (11 %) patients who reported an event, three were considered serious using ICH guidelines and included two deaths and one hospitalization; none were classified as related to SLD PQ (Table 4). In all three serious cases, the underlying clinical diagnosis was unclear and reported as a multitude of symptoms. One death was attributed to the patient’s pre-existing immune-compromised condition. This patient, presenting with fever, chills, diarrhoea and vomiting, was diagnosed with uncomplicated *P. falciparum* malaria, testing positive by rapid diagnostic test (RDT) and microscopy. He was admitted to the hospital as a precaution to monitor his diarrhoea. The following day he was enrolled and treated with AL plus SLD PQ, and panadeine. The diarrhoea continued throughout the day. He was confused. Diclofenac was added to his treatment. Two days after symptom onset, the patient died. Another patient presenting with complaints of weakness, jaundice, and coughing, was prescribed AL based on signs and symptoms, despite testing negative for malaria by RDT and microscopy. When the patient returned to the clinic to pick up his AL treatment (two days after event onset), the patient was incorrectly enrolled into the study and treated with AL plus SLD PQ. Despite anti-malarial therapy, the patient’s condition continued to deteriorate. Upon active follow-up by telephone, the study nurses learned of the patient’s death. A verbal autopsy suggested that the patient’s health had deteriorated to the point where the patient was unable to pass urine. He died within 10 days of initial presentation. One patient, with complaints of diarrhoea, vomiting, headache, and dizziness, was admitted and hospitalized one day after treatment with SLD PQ but recovered within 48 h of onset.

**Table 4:** Detailed summary of adverse events and serious adverse events* as reported after exposure to SLD PQ together with AL

Sex	Age (years)	Baseline Hb (g/dL)	Follow-up Hb (g/dL)	Adverse event	Severity grade	Actions taken	Outcome
Male	32	14.9	13.2	Dark urine	Moderate	Urinalysis and CBC	Recovered
Male	28	15.7	15.8	Dark urine	Moderate	None	Recovered
Female	63	12.6	9.6	Unwell (worsening post-dose)	Moderate	None	Unknown
Female	18	9.2	–	Anaemia, back pain, fever, and vomiting after meals	Moderate	Prescribed drug	Recovered
*Female	31	14.1	12.5	Diarrhoea, vomiting, headache, and dizziness	Moderate	Hospitalization	Recovered
Male	5	9.3	8.0	Dark urine (grade: 8)	Severe	None	Recovered
Female	30	14.9	13.6	Nausea	Moderate	None	Recovered
*Male	60	12.8	–	Fever, chills, diarrhoea and vomiting	N/A	Prescribed drug	Fatal
Male	44	9.8	11.2	Dark urine (grade: 5)	Mild	None	Recovered
*Male^a^	28	19.5	–	Weak, jaundice, coughing	N/A	Prescribed drug	Fatal
Female	3	10.5	11.6	Stomach cramps	Mild	Unknown	Recovered

Dashes represent missing values

*AL* artemether-lumefantrine, *CBC* complete blood count, *Hb* haemoglobin, *N/A* not applicable, *SLD PQ* single low-dose primaquine

^a^Received anti-malarial treatment despite testing negative for malaria by rapid diagnostic test and microscopy

None of the patients vomited in the 30 min following drug administration. The most frequently reported events in all 11 patients were dark urine (36 %), vomiting (27 %), fever (18 %), and diarrhoea (18 %). Four non-serious adverse events were considered definitely, probably or possibly related to SLD PQ. Of the non-serious adverse events, all recovered except for one whose outcome remains unknown despite multiple telephone attempts at follow-up. This patient, following a diagnosis of vivax malaria confirmed by microscopy was referred to one of the study facilities and received the recommended higher dose of PQ (30 mg). Following treatment, the patient reported malaise and worsening of symptoms.

### Dissemination and impact

**Fig. 3:**
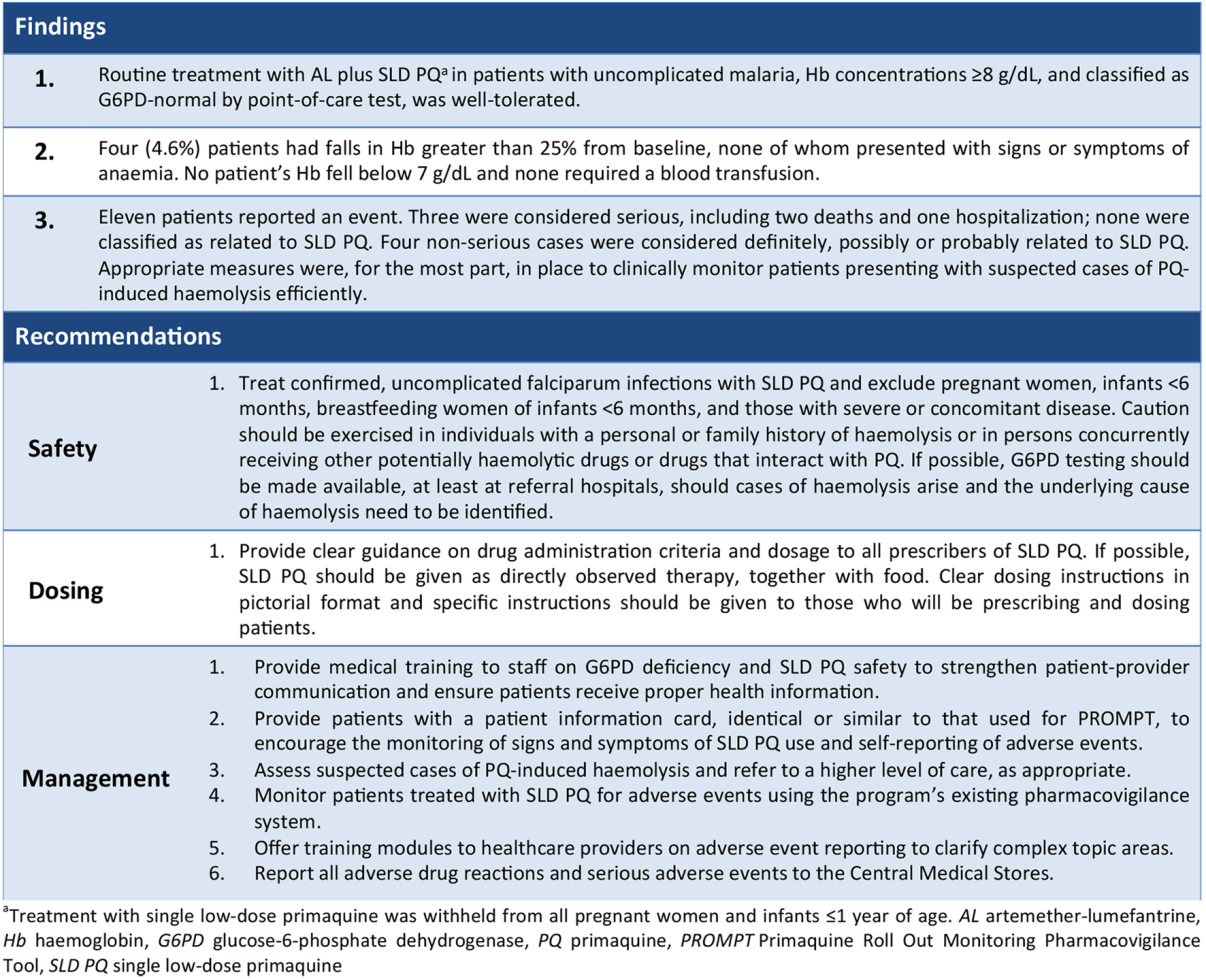
Summary of key findings and recommendations

Key findings from the safety assessment were disseminated and recommendations were made (Fig. 3) to key stakeholders during the NMCP’s Malaria Elimination Advisory Group General Committee meeting in January 2016. Results and recommendations on safety, dosing, and the management of adverse events were shared to prepare for countrywide adoption of the WHO recommendation. Regional trainings launching the new diagnosis and treatment guidelines followed. As of December 2015, SLD PQ has been available at all three levels of Swaziland’s health system. This includes both public and private clinics, health centres, and referral hospitals, making Swaziland the first country in sub-Saharan Africa to rollout SLD PQ for malaria elimination.

## Discussion

This study is the first to demonstrate the feasibility and acceptability of performing active safety monitoring using a simple pharmacovigilance tool in those prescribed SLD PQ therapy for the treatment of *P. falciparum* malaria. Results suggest that PROMPT achieved its objective and successfully yielded standardized safety data on the range and frequency of adverse events, including haematologic changes, experienced after SLD PQ therapy. The data in turn contributed to a limited but growing local evidence base and offered the program insight prior to countrywide adoption of WHO policy. These findings are supported by the following observations.

Firstly, PROMPT promoted and facilitated the capture of treatment emergent adverse events after initiation of SLD PQ therapy with a known denominator of the number of people exposed to the drug (n = 102). Calculating adverse drug reaction frequencies or incidence rates to examine risk for particular treatments is limited with current systems that rely primarily on passive, spontaneous reporting [14]. Importantly, patients with signs or symptoms suggestive of PQ-induced haemolysis or other serious events were, for the most part, promptly identified and monitored within a week of drug administration. Despite two deaths, unrelated to the intake of SLD PQ, and one patient who was lost to follow-up after receiving a higher dose of PQ (30 mg) for vivax malaria, all *P. falciparum* patients who presented with an adverse event recovered. Moreover, repeat haematologic testing ensured that clinically important drops in haemoglobin (i.e., fractional drops in haemoglobin ≥25 % from baseline or haemoglobin concentrations <7 g/dL post-treatment) were detected. No patient’s haemoglobin fell below 7 g/dL and none required a blood transfusion.

Second, this study showed high adherence (>90 %) to visits on or near day 7 and demonstrated the acceptability of this approach among trained nurses. The use of simple approaches—real-time electronic data collection, SMS text reminders, follow-up phone calls, and patient information cards—encouraged patients to return to clinics and helped ensure the timely reporting of safety data to a central repository. This is in line with prior research illustrating how sensitization and training of health workers and consumers can be strong incentives for reporting and adherence to scheduled visits [22]. Furthermore, there is growing evidence on the impacts of mobile health (mHealth) interventions on health outcomes in low- and middle-income countries [23]. Pharmacovigilance and post-marketing surveillance of the safety and quality of medicines has been proposed as one of six areas within malaria control where the use of text messaging technology could improve routine delivery of health services in Africa [24]. Some settings may however not have the resources to support pharmacovigilance strategies that rely on advanced technologies, active follow-up, and the literacy of participants. Alternative strategies that are feasible and appropriate using paper forms with less-intense monitoring and patient information cards formatted for illiterate individuals using pictorial methods should be adopted.

This study did, however, face challenges that are inherent to establishing pharmacovigilance systems with reliable reporting processes [14]. Underreporting, likely due to a lack of understanding as to what constitutes an adverse event, appears to have been higher than anticipated. Results reveal that patients and providers may have selectively reported adverse events potentially associated with SLD PQ and suggestive of haemolysis (e.g., dark urine and paleness/jaundice). In addition, there was no baseline assessment of symptoms in order to reduce the workload for healthcare providers. Thus, while procedures specified that an abnormality present at baseline should not be recorded unless it increased in severity, doing so relied on patients recalling exactly when symptoms started at least one week prior. Recall is usually better in the short-term [25]; however, certain events may have been dismissed, especially those less severe, unexpected or thought to be a clinical manifestation of malaria, despite having an onset post-SLD PQ administration and which should, by definition, have been reported. Methods for eliciting information could have also varied and influenced the detection of adverse events. Future research should explore factors that influence patterns of reporting to better accommodate these challenges.

There are limitations to the study also worth noting. Sampling was restricted to two hospital facilities in a low transmission African setting in relatively healthy participants with haemoglobin concentrations of 8 g/dL or greater, and no enrolled patients were G6PDd, which may limit the generalizability of our findings. Furthermore, by not enrolling any G6PDd individuals, the targeted G6PDd sample size was not achieved. This could be due to a low prevalence of G6PD deficiency in this malaria-infected population or due to a multitude of factors that affect the performance of G6PD tests. Recent haemolytic events can leave patients with a large proportion of young red blood cells in circulation. These cells have high G6PD enzyme function and can obscure correct diagnosis and produce false normal results. In addition, borderline colourimetric results can produce subjective and variable readings [26]. For these reasons, confirmatory testing would be required to avoid misclassification of results and examine this in the future. The qualitative rapid test can only reliably identify completely deficient phenotypes.

This study also relied on the views of four trained nurses to produce opinions on tool acceptability thus restricting the breadth of opinions and experiences captured. Nonetheless, perceptions did demonstrate promise that PROMPT would be well received on a larger scale. Additional interviews among end users would allow us to capture a broader range of views in the future.

Expanding PROMPT’s use beyond Swaziland presents an opportunity to address these limitations and improve the systematic collection of safety data where data are needed. Data that include G6PDd participants are being collected from four additional countries across Asia and Africa (Bangladesh, Senegal, Sudan and Tanzania) with differing levels of risk associated with PQ therapy. As data from these sites increases in size, it will be possible to pool individual patient data, directly compare, and determine the risks of PQ therapy with ever-greater accuracy. These new insights could serve to inform the highest safe dose of SLD PQ in G6PDd individuals that is critical to defining the therapeutic dose range and guiding appropriate dosing regimens [27].

PROMPT could also extend to support pharmacovigilance activities under diverse contexts and in select populations. For example, subjects could be followed up at additional time points (e.g., days 14 and 28) to assess outcomes after PQ treatment of longer duration for *P. vivax* or *P. ovale*. Women of childbearing age inadvertently exposed to PQ in very early, unsuspected pregnancy could also be identified at around 3 months and prospectively followed at regular intervals (e.g., every 3 months or so until several weeks post-natal) to assess pregnancy and birth outcomes. Data on the use of PQ in pregnancy remains limited and further study on the safety of PQ in pregnancy are needed. Finally, these tools could be expanded beyond PQ to other drugs for a variety of other diseases.

As access to SLD PQ increases, a larger evidence-base to support the safety of the latest WHO recommendation will be needed. Several countries in the Asia Pacific, including Bhutan, Indonesia, Malaysia, Myanmar, Thailand, the Philippines, and Vietnam include SLD PQ in their *P. falciparum* treatment guidelines, albeit in different doses and schedules. And demand for its use is growing rapidly in parts of Africa with Botswana, Ethiopia, Namibia, South Africa, Swaziland, Zanzibar, and Zimbabwe who have written SLD PQ into national strategic plans or treatment guidelines for falciparum malaria [28]. Although evidence suggests that the risk of haemolytic toxicity in G6PDd individuals treated with a 0.25 mg/kg single dose of PQ for falciparum malaria is low [13], no medicine is absolutely free from risk. Obtaining safety data on the use of SLD PQ in settings that extend beyond small and selected populations that are characteristic of randomized controlled clinical trials to reflect real-life usage in programmatic settings will help reduce uncertainties that surround the safety of SLD PQ. PROMPT holds potential for becoming a practical tool for generating locally acquired safety data and supporting pharmacovigilance in resource-limited settings, however, wider adoption is needed to further evaluate and refine its use.

## Additional files

10.1186/s12936-016-1410-7 Urine colour scale designed to assess the degree of haemoglobinuria. Dark-coloured urine with a colourimetric of 5 or above was considered evidence of haemoglobinuria [18]. With permission from the NEJM.
10.1186/s12936-016-1410-7 Example of a patient information card in English.
10.1186/s12936-016-1410-7 Sample size guide.

## Abbreviations

ACT: artemisinin-based combination therapy
AL: artemether-lumefantrine
API: active pharmaceutical ingredient
NMCP: National Malaria Control Programme
G6PD: glucose-6-phosphate dehydrogenase
G6PDd: G6PD-deficient
Hb: haemoglobin
IQR: interquartile range
mHealth: mobile Health
MoH: Ministry of Health
*P. falciparum*: *Plasmodium falciparum*
Pfs25 QT-NASBA: Pfs25 RNA-based quantitative nucleic acid sequence-based amplification
PQ: primaquine
PROMPT: Primaquine Roll Out Monitoring Pharmacovigilance Tool
RDT: rapid diagnostic test
SD: standard deviation
SLD PQ: single low-dose primaquine
WHO: World Health Organization
