# Supplementary material for: Development of a pharmacovigilance safety monitoring tool for the rollout of single low-dose primaquine and artemether-lumefantrine to treat Plasmodium falciparum infections in Swaziland: a pilot study
Source: Malar J. 2016 Jul 22;15:384. doi: 10.1186/s12936-016-1410-7 (PMC4957931; doi:10.1186/s12936-016-1410-7)

**Figure S1**. Urine colour scale designed to assess the degree of haemoglobinuria. Dark-coloured urine with a colourimetric of 5 or above was considered evidence of haemoglobinuria (18, Panel A). With permission from the NEJM.


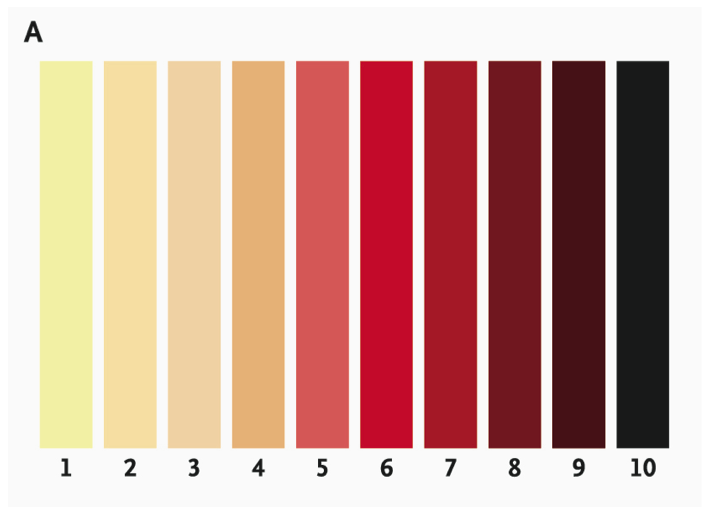

Supplement: Supplementary file 1 — 10.1186/s12936-016-1410-7 Urine colour scale designed to assess the degree of haemoglobinuria. Dark-coloured urine with a colourimetric of 5 or above was considered evidence of haemoglobinuria [18]. With permission from the NEJM. [file 12936_2016_1410_MOESM1_ESM.docx]
