# Supplementary figures and images for: Development of a pharmacovigilance safety monitoring tool for the rollout of single low-dose primaquine and artemether-lumefantrine to treat Plasmodium falciparum infections in Swaziland: a pilot study
Source: Malar J. 2016 Jul 22;15:384. doi: 10.1186/s12936-016-1410-7 (PMC4957931; doi:10.1186/s12936-016-1410-7)

**Figure S2.** Example of a patient information card in English.

*
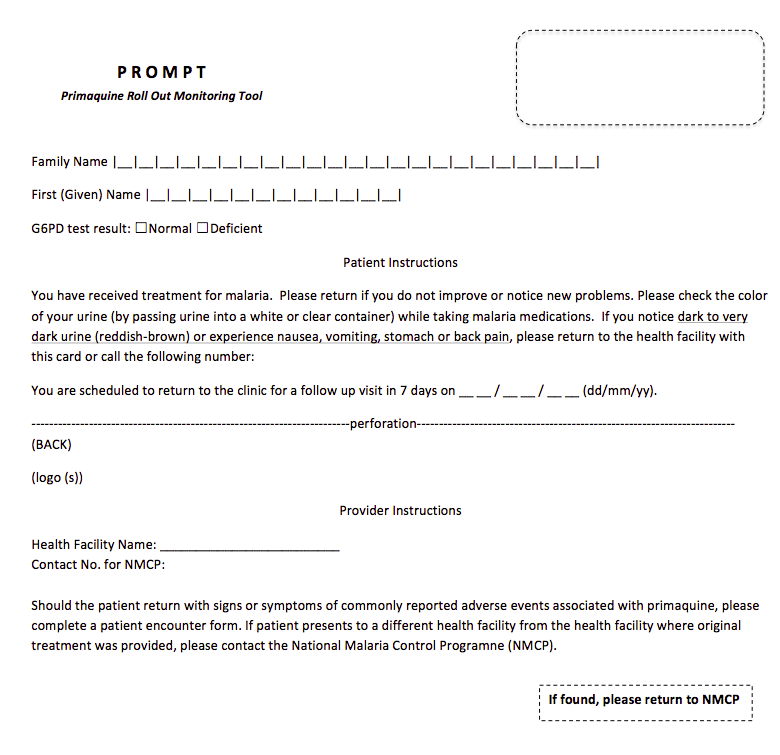
*

Supplement: Supplementary file 2 — 10.1186/s12936-016-1410-7 Example of a patient information card in English. [file 12936_2016_1410_MOESM2_ESM.docx]
