# Supplementary material for: Development of a pharmacovigilance safety monitoring tool for the rollout of single low-dose primaquine and artemether-lumefantrine to treat Plasmodium falciparum infections in Swaziland: a pilot study
Source: Malar J. 2016 Jul 22;15:384. doi: 10.1186/s12936-016-1410-7 (PMC4957931; doi:10.1186/s12936-016-1410-7)
Supplement: Supplementary file 3 — 10.1186/s12936-016-1410-7 Sample size guide. [file 12936_2016_1410_MOESM3_ESM.docx]

# Sample size guide

To determine what constitutes a “sufficient” sample size for the proposed pilot, a number of considerations must be taken into account.

## For settings where the patient’s G6PD status is known prior to treatment with primaquine:

**Table S1.** Number of persons needed to treat to have sufficient power to detect a given within-person percent reduction in haemoglobin

| Minimum percent reduction in haemoglobin that you want to be able to detect after treatment with primaquine | Number of G6PD-deficient persons needed to treat |
| --- | --- |
| 15% | 11 |
| 20% | 7 |
| 25% | 5 |
| 50% | 3 |

Detects a within-person change in haemoglobin between Days 0 and 7 with 80% power, and a significance level of 0.05 (one-sided), assumes mean of 10 g/dL before treatment, a standard deviation of the within-person change in haemoglobin of 2 g/dL, and no loss to follow-up

The sample sizes in **Table S1** assume that G6PD screening is available at the individual level. If the program wants to know whether primaquine treatment is associated with a 25% or greater drop in haemoglobin (e.g., a drop from 10 g/dL to 7.5 g/dL or lower), the program would need to measure the change in haemoglobin in at least 5 G6PD-deficient individuals before and after treatment with primaquine. A sample of 5 G6PD-deficient individuals would give the program 80% power to detect a 25% or greater average within-person drop in haemoglobin as statistically significant. Programs that want to be able to detect a smaller average within-person drop in haemoglobin as significant, for example a 15% drop, would need to test a larger number of people (**Table S1**).

## For settings where the patient’s G6PD status is unknown prior to treatment with primaquine:

In settings where individuals’ G6PD status is unknown and G6PD screening is unavailable, programs can use the population prevalence of G6PD deficiency to estimate the number of individuals that need to be treated with primaquine and have pre-post treatment haemoglobin measurements. Let’s assume that the population prevalence of G6PD deficiency is 1% and the program wants to be able to detect a 25% or greater within-person drop in haemoglobin following treatment with primaquine. Using **Table S1**, we know that the program would need to measure the change in haemoglobin in at least 5 G6PD-deficient individuals before and after treatment with primaquine. Assuming G6PD-deficient individuals are randomly distributed among the population, the program would need to measure the change in haemoglobin among at least 500 individuals of unknown G6PD status before and after treatment with primaquine (**Table S2**). By evaluating 500 individuals, the program can assume that 1% of those 500, or 5 people, were G6PD-deficient. The numbers presented in **Table S2** are derived by dividing the number needed to treat in Table S1 by the estimated population prevalence of G6PD (5/0.01 = 500).

**Table S2.** Number of persons needed to treat to have sufficient power to detect a given within-person percent reduction in haemoglobin

|  | Numbers of individuals needed to treat with primaquine  by the estimated population prevalence of G6PD deficiency | | | |
| --- | --- | --- | --- | --- |
| Minimum percent reduction in haemoglobin that you want to be able to detect after treatment with primaquine | **1%** | **5%** | **10%** | **15%** |
| 15% | 1,100 | 220 | 110 | 74 |
| 20% | 700 | 140 | 70 | 47 |
| 25% | 500 | 100 | 50 | 34 |
| 50% | 300 | 60 | 30 | 20 |

Detects a within-person change in haemoglobin between Days 0 and 7 with 80% power, and a significance level of 0.05 (one-sided), assumes mean of 10 g/dL before treatment, a standard deviation of the within-person change in haemoglobin of 2 g/dL, and no loss to follow-up

## For settings where the patient’s G6PD status is known prior to treatment with primaquine with 25% loss to follow-up:

One further consideration might be the loss to follow-up of individuals who are treated with primaquine. It may not be reasonable to expect that all people who are treated with primaquine will return 7 days later for a post-treatment haemoglobin measurement. Using the example above, if we assume that the program will lose up to 25% of individuals between the time of treatment and the time of their follow-up haemoglobin measurement, programs may want to treat and evaluate an additional 25% of people. For example, if the program plans to measure the change in haemoglobin in at least 5 G6PD-deficient individuals before and after treatment with primaquine, the program would want to treat 7 individuals to ensure that 5 have both a pre- and post-treatment haemoglobin measurement. The numbers in **Table S3** are derived by dividing the number of individuals needed by 1 minus the estimated loss to follow-up. For example, 5/(1-0.25) = 6.7, or 7 G6PD-deficient individuals. Similar calculations are done in **Table S4** for settings where G6PD status is unknown.

**Table S3.** Number of persons needed to treat to have sufficient power to detect a given within-person percent reduction in haemoglobin assuming 25% loss to follow-up

| Minimum percent reduction in haemoglobin that you want to be able to detect after treatment with primaquine | Number of G6PD-deficient persons needed to treat |
| --- | --- |
| 15% | 15 |
| 20% | 10 |
| 25% | 7 |
| 50% | 4 |

Detects a within-person change in haemoglobin between Days 0 and 7 with 80% power, and a significance level of 0.05 (one-sided), assumes mean of 10 g/dL before treatment, a standard deviation of the within-person change in haemoglobin of 2 g/dL, and up to 25% loss to follow-up

## For settings where the patient’s G6PD status is unknown prior to treatment with primaquine with 25% loss to follow-up:

**Table S4.** Number of persons needed to treat to have sufficient power to detect a given within-person percent reduction in haemoglobin, assuming 25% loss to follow-up

|  | Numbers of individuals needed to treat with primaquine  by the estimated population prevalence of G6PD deficiency | | | |
| --- | --- | --- | --- | --- |
| Minimum percent reduction in haemoglobin that you want to be able to detect after treatment with primaquine | **1%** | **5%** | **10%** | **15%** |
| 15% | 1,500 | 300 | 150 | 100 |
| 20% | 1000 | 200 | 100 | 67 |
| 25% | 700 | 140 | 70 | 47 |
| 50% | 400 | 80 | 40 | 27 |

^1^Detects a within-person change in haemoglobin between Days 0 and 7 with 80% power, and a significance level of 0.05 (one-sided), assumes mean of 10 g/dL before treatment, a standard deviation of the within-person change in haemoglobin of 2 g/dL, and up to 25% loss to follow-up
